# Oncogenic Viruses as Entropic Drivers of Cancer Evolution

**DOI:** 10.3389/fviro.2021.753366

**Published:** 2021-11-15

**Authors:** Italo Tempera, Paul M. Lieberman

**Affiliations:** Program in Gene Expression and Regulation, The Wistar Institute, Philadelphia, PA, United States

**Keywords:** KSHV, plasticity, hepatitis B virus, epigenetic, cancer, Merkel cancer, HPV–human papillomavirus, EBV–Epstein-Barr virus

## Abstract

Viral infection is an indisputable causal factor for nearly 17% of all human cancers. However, the diversity and complexity of oncogenic mechanisms raises new questions as to the mechanistic role of viruses in cancer. Classical viral oncogenes have been identified for all tumor-associated viruses. These oncogenes can have multiple oncogenic activities that may or may not be utilized in a particular tumor cell. In addition, stochastic events, like viral mutation and integration, as well as heritable host susceptibilities and immune deficiencies are also implicated in tumorigenesis. A more contemporary view of tumor biology highlights the importance of evolutionary forces that select for phenotypes better adapted to a complex and changing environment. Given the challenges of prioritizing singular mechanistic causes, it may be necessary to integrate concepts from evolutionary theory and systems biology to better understand viral cancer-driving forces. Here, we propose that viral infection provides a biological “entropy” that increases genetic variation and phenotypic plasticity, accelerating the main driving forces of cancer cell evolution. Viruses can also influence the evolutionary selection criteria by altering the tumor microenvironment and immune signaling. Utilizing concepts from cancer cell evolution, population genetics, thermodynamics, and systems biology may provide new perspectives on viral oncogenesis and identify novel therapeutic strategies for treating viruses and cancer.

## INTRODUCTION

Viruses have well-established causal roles in numerous human and animal cancers, collectively responsible for almost one fifth of all cancers (1, 2). Viral associated cancers are a special case of cancer biology and virology. To date, there are seven human viruses with strong epidemiological links to human cancers. These include members of the high-risk human papillomavirus (HPVs), hepatitis viruses B and C (HBV and HCV), human gammaherpesviruses (HHV4/Epstein-Barr Virus (EBV) and HHV8/Kaposi’s Sarcoma-Associated Herpesvirus (KSHV), Merkel cell polyomavirus (MCPyV), and human T-cell leukemia virus I (HTLV-1). These oncoviruses represent members of vastly different families of virus, including DNA, RNA and retroviridae. Despite this species diversity, these oncoviruses are thought to share common features that enable them to drive cancer. Oncoviruses usurp key cellular pathways important for the control of cell growth and metabolism. However, many non-cancer-causing viruses perturb these pathways and have similar viral-host interactions. Consequently, it is not fully understood what features confer viruses with oncogenic potential in human populations.

**FIGURE 1 |:**
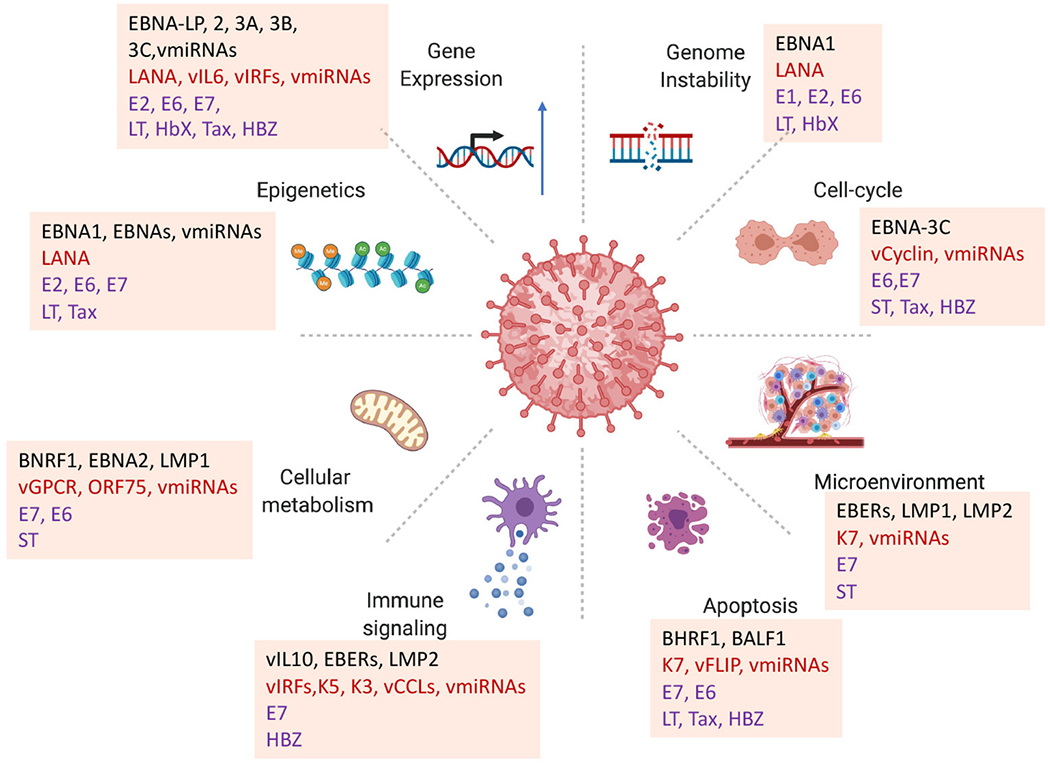
Diversity of viral oncogenic mechanisms. Viral oncogenes have been shown to perturb multiple hallmarks of cancer. How each of these viral-host interactions contribute to carcinogenesis in a particular viral cancer is a challenge for identifying primary driving forces of viral cancers. Examples highlighted from EBV proteins EBNA-LP, BNA1, EBNA2, EBNA3A, 3B, 3C, BNRF1, BZLF1; KSHV LANA, vGPCR, vCyclin, vIL6, ORF75; HPV E6, E7; MCPyV LT, ST; HBV HbX; HTLV-1 Tax, HBZ.

Oncogenic viruses perturb numerous cellular pathways described as the hallmarks of cancers (3, 4). As expected, viral-associated cancer pathways can be readily superimposed on these cancer hallmarks (5) (Figure 1). And while the pathways of viral carcinogenesis are ultimately cellular, viruses do provide foreign genomes and gene products that create new interactions and pathways for oncogenesis. How do these viral products and viral-specific pathways work coordinately over time to overcome the many barriers to cellular carcinogenesis? What makes these seven viruses different from their non-oncogenic relatives? Here, we suggest that oncogenic viruses are unique in their ability to increase the adaptability and evolvability of infected cells, and that multiple perturbations over time enable formation of cancer cell fate choices. We suggest that a more in-depth knowledge of virus-host interactions over the time-course of cancer evolution will provide a more complete understanding of viral oncogenesis.

## A PLETHORA OF ONCOGENIC MECHANISMS

A major challenge in the field of viral oncology, and cancer biology in general, is the very large number of mechanisms and pathways that contribute to carcinogenesis. While early studies focused on one or a few viral oncogenes, we now know that viruses can promote cancers through a much greater diversity of mechanisms and pathways. Many of these mechanisms can be well-defined in the context of a particular tumor type or tissue environment. However, the diversity of mechanisms confounds the identification of single causal agent or event. Given the abundance of potential and actual mechanisms, a new challenge arises to both understand the impact of each oncogenic event and the accumulation of multiple distinct oncogenic events over time or in the context of a transient stress challenge. Here, we provide a brief overview of some classic mechanisms of viral oncogenesis, highlighting many from the human gammaherpesviruses, and argue that the multiplicity of these mechanisms makes sense only in the context of cancer cell evolution and systems biology.

### Classical Viral Oncogenes Are Not Sufficient for Cancer

Viruses have been shown to induce tumor formation through numerous and diverse mechanisms (5). From the pioneering studies of Peyton Rous in 1920s, retroviruses were found to encode oncogenes, like v-src, v-myc, v-ras, which could potently transform normal cells to grow as tumors in animal models (6). Several of these viral oncogenes were subsequently recognized as host genes captured by viral recombination and their oncogenicity due to combinations of activating mutations and deregulated expression in infected cancer cells. DNA tumor viruses were found to encode novel oncoproteins, such as SV40 T-antigen, adenovirus E1A and E1B, and papillomavirus E6 and E7 that interact with and disable cellular tumor suppressor proteins, such as p53 and Rb (7). While these viral oncogenes may be necessary for transformation in laboratory models, they are not sufficient for tumor formation in the overwhelming majority of natural infections. In addition, some viruses, such as adenovirus, encode potent inhibitors of cellular tumor suppressors p53 and Rb, but rarely associate with human cancers (8). This is consistent with the observation that most oncogenic viruses typically cause benign infections, that only rarely lead to cancer.

### Multiple Host Targets of Viral Oncogenes

Pioneering studies identified a few cellular targets universally exploited by tumor viruses, such as p53 DNA damage surveillance and Rb cell cycle control. However, it is now known that subversion of these targets are not sufficient for viral tumorigenesis, and that additional and alternative host proteins and pathways are targeted by viral oncogenes (Figure 1). For example, the small viral oncoprotein E7 is well known to bind and degrade Rb (9), but can also interact with the Rb-associated DREAM complex (10), phosphatase PTPN1(11), histone modifying enzyme HDACs (12), stem cell promoting factors APH1B and OCT4 (13, 14), and Cullin2 to stabilize APOBEC3a (15). In addition, E7 can cooperate with another viral oncoprotein E6 to activate hTERT to overcome replicative senescence (16, 17). Viral subtypes, as well as host cell type can determine whether these different interactions are oncogenic, further demonstrating the diversity of targets for one small viral oncoprotein and its potential effects on different cancer pathways. Similarly, the MCPyV small T antigen can interact with MYCL and EP400 to alter chromatin and transcription regulatory networks implicated in cell lineage control (18), 4EBP1 affecting translational control (19), protein phosphatase 2A to affect ubiquitin ligases (20), the F-box proteins FBW7 to activate NF-kB signaling (21, 22), as well as binding iron-sulfur clusters (23). This promiscuous multitasking is likely to be a general feature of viral oncoproteins that target multiple cellular proteins and pathways.

Larger DNA tumor viruses, such as EBV and KSHV, encode numerous viral genes implicated in oncogenesis. Many of these oncogenes target pathways important for the tissue-specific functions of the host cell. EBV encodes two membrane oncoproteins, LMP1 and LMP2, that cooperate to immortalize primary B-lymphocytes by mimicking B-cell receptor and CD40 co-receptor (24, 25). LMP1 interacts with multiple TRAFs and TRADDs to activate NF-kB pathways (26), but can also interact with other proteins involved in membrane vesicle formation (27). LMP2 can interact with several different src-family kinases (28). EBV also encodes 6 nuclear antigens, EBNA-LP, 1, 2, 3a, 3b, and 3C, that are all implicated in oncogenic mechanisms (29). EBNA2 is absolutely required for B-cell immortalization *in vitro* (30), and natural mutations in EBNA2 correlate with B-cell transformation activity (31). However, some EBV associated tumors fail to consistently express EBNAs and LMPs. Like EBV, KSHV also encodes candidate oncogenes, including the nuclear antigen LANA that can bind host chromatin and alter p53 and Rb function, vGPCR that can induce endothelial tumors in transgenic mouse models, vCyclin that can drive cell cycle progression, vFLIP and K12 that can activate NF-kB and STAT3 signaling (32). In addition to these viral encoded proteins, both EBV and KSHV have numerous non-coding RNAs implicated in oncogenesis. EBV small non-coding RNA EBERs can interact with transcription factor PAX5 (33), ribosomal protein L22 (34), TLR receptors (35), and provide paracrine signals through exosome transmission (36). Numerous viral miRNAs target oncogenic pathways implicated in EBV and KSHV carcinogenesis (37, 38). EBV miRNA are highly expressed in tumors, especially EBV-associated gastric carcinomas (EBVaGC) along with other non-coding RNAs that arise from the same genomic locations (BARTs) and have additional oncogenic potential (39, 40). Remarkably, these viral genes are expressed at variable levels and heterogeneously in most viral-associated cancers, further confounding the problem of complexity and diversity of viral oncogenic mechanisms.

### A Growing List of Viral Oncogenic Mechanisms

#### Inhibition of Apoptosis

Resistance to programmed cell death, particularly apoptosis, is among the most fundamental hallmarks of cancer and viral infection. Viruses provide numerous mechanisms to resist apoptosis (41–43). For example, EBV encodes two viral proteins, BHRF1 and BALF1, dedicated to inhibition of the Bcl2 family of pro-apoptotic factors. BHRF1 and BALF1 have some overlapping, but not completely redundant activities in the inhibition of programmed cell death mediated by Bcl2 (44, 45). In addition, EBV encodes multiple miRNAs that target the pro-apoptotic genes BIM (46) and Puma (47). KSHV also encodes numerous genes directed at the disruption of apoptosis (42), including a Bcl2 homologue ORF16 that may have mitochondrial and nuclear functions required for viral reactivation and lytic replication (48). MCPyV large T protein enhances BIRC5/survivin mRNA and protein expression to prevent caspase-mediated apoptosis (49). HBV HbX protein has been shown to have a BH3-like domain that interacts with Bcl2 and Bcl-xL to prevent apoptosis during viral replication (50). HCV non-structural protein NS5A can attenuate apoptosis by enhancing GRP78 expression and reducing ER-stress (51). HTLV-1 Tax suppresses transcription of pro-apoptotic genes Bid and Bim, while activating expression of pro-survival Bcl-2 members (52). In general, oncoviruses demonstrate numerous and diverse anti-apoptotic mechanisms, often encoding multiple, partially redundant viral genes that may be expressed heterogeneously in tumors.

#### Reprogramming Host Metabolism

Cancer cells frequently undergo a metabolic shift to aerobic glycolysis (Warburg effect) and utilize alternative metabolites, such as glutamine and serine for energy production and macromolecular biosynthesis (53). Oncogenic viruses can reprogram cellular metabolism in various ways (54). Overexpression of both HPV16 E6 and E7 promote glucose metabolism through activation of glucose transporter 1 (55). E6 was shown to stabilize HIF1A induced Warburg effect during hypoxia in keratinocytes (56, 57). E7 can bind and inhibit pyruvate kinase M2 to promote glycolysis (58). MCPyV small T has been shown to increase glucose consumption and lactate production indicative of Warburg aerobic glycolysis (59). These changes correlate with transcriptomic changes in hypoxia, AMPK activation, and mTOR signaling. EBV infection of resting B-cells induces a hyperproliferative state that is rate limited by nucleotide metabolism (60), and EBNA2 activates a myc-dependent metabolic program to increases amino acid and nucleotide metabolism during hyperproliferation (61). EBNA2 also activates SREBP2 to promote lipid biosynthesis and fatty acid metabolism (62). KSHV miRNAs induce a metabolic shift from OXPHOS to glycolysis (63). Thus, oncogenic viruses shift cellular metabolism through multiple factors and pathways to promote infected cell fitness, similar to cancer cells.

#### Modulation of the Cellular Microenvironment

Viral-infected cells and associated tumors thrive in harsh microenvironments that reinforce viral-infected and cancer cell selection, requiring Warburg metabolism and adaptation to low oxygen and acidification (64, 65). Variations in oxygenation due to competitive crowding or vascular insufficiency can have dramatic effects on viral gene expression and cellular stress response (65). Hypoxia plays a central role in KS tumorigenesis and regulating KSHV latency (66, 67). Hypoxia inducible factors (HIF1A) can modulate KSHV oncogenes, including vIL6 and vGPCR to upregulate VEGF and angiogenesis (68, 69). Hypoxia has a strong immunosuppressive effect (70), and oncogenic viruses may induce pseudohypoxia to escape immune recognition (71). Viral induced Warburg effect can also be immunosuppressive by competing with immune cells for glucose and oxygen consumption (72). Hypoxia is known to induce EBV lytic cycle genes that have pro-survival and immune modulatory functions. EBV BRLF1 can inhibit interferon response genes IRF3 and IRF7 and interferon production (73) while BZLF1 can inhibit interferon gamma and TNFβ signaling pathways (74, 75). EBV immediate early protein BZLF1 can also induce SOCS3 to inhibit cytokine signaling (76), and the viral kinase BGLF4 can degrade TLR9 mRNA (77). Oncogenic viruses also reprogram the tumor microenvironment through production of extracellular vessicles (EV) that transmit cargo to neighboring cells (63, 78). EBV positive NPC produce EVs that transfer viral miRNAs and oncoproteins, such as LMP1, to neighboring cells (36, 79) causing a microenvironment selective for infected cell persistence (80) and immune suppression through recruitment of regulatory T-cells (81). Thus, virus infection can alter the microenvironment to promote selection of viral-infected cells that survive at the expense of uninfected and non-transformed cells.

#### Attenuation of Host Immune Control

In Darwinian terms, viruses and cancer are most limited by the predatory function of the immune system. Viral associated cancers are particularly adept at modulating immune surveillance, and are most virulent in immunosuppressed conditions, such as HIV-AIDS and solid-organ transplants. HPV and EBV associated tumors upregulate T-cell checkpoint proteins PD-L1 and PD-L2, as well as the CTLA-4 immunosuppressive pathways (82). Multiple different mechanisms act on this pathway. For EBV, EBNA2 can down regulate miR-34a to upregulation of PD-L1 in lymphoid cancers (83). LMP1 activates PD-L1 through interferon gamma pathway in NPC (84). LMP1 activates PD-L1 through NF-kB pathway in NKTCL (85). HPV E5 protein suppresses HLA expression and immune recognition of infected tumor cells, rendering them resistant to checkpoint immunotherapy (86). HBV sAg binds to SIGLEC-3 (CD33) on myeloid cells to induce immunosuppression (87). HCV core protein interaction with cellular gC1qR can modulate macrophage cytokines to restrict immune targeting to HCC (88). Clearly, diverse and novel viral mechanisms function to disable the immunological barriers to cancer.

#### Transcriptional Reprogramming

Perturbations in transcription factor and gene regulatory networks are also hallmark changes in cancer. Viral immediate-early genes and oncogenes frequently target transcription factors and transcription factor networks that are fundamental to host cell differentiation and identity. E6, E7, and T-antigens are known to interact with cellular transcription factors, such as p53 and the Rb-family complexes. EBV and KSHV encode numerous other nuclear factors that alter transcription control, and these viral factors have been implicated in viral carcinogenesis. All six EBV-encoded EBNAs interact with host transcriptional regulators to perturb regulatory networks in distinct and complex ways. EBNA2 can interaction with B-cell regulatory factors RBPJ, EBF1, RUNX1, and PU.1 to affect cooperative DNA binding site selection (89) as well as facilitate formation of super-enhancers, such as found at the cMyc locus (90, 91). EBNA1 can activate transcription of the EBNA2 gene, while EBNA2 can auto-activate its own transcription along with that of EBNA3Cs and LMPs to change the EBV viral gene regulatory network. Similarly, these factors cooperate to regulate expression of host cell genes including the repression of tumor suppressor genes, like BIM and p16, by EBNA3C (92, 93). In contrast, KSHV encodes one major nuclear protein LANA that can affect viral and cellular transcription and chromatin structure through multiple mechanisms including direct binding to core histones H2A/H2B through its N-terminus and to GC-rich DNA through its C-terminal domain (94, 95). KSHV also encode Interferon Regulatory Factors (vIRFs) that can alter transcriptional control of cellular IRFs, but these viral factors are not typically expressed in most KSHV-associated tumor cells, unless lytic reactivation occurs (96). EBV and KSHV miRNAs and longer non-coding RNAs can also impact cellular and viral regulatory networks (97). All other oncogenic viruses have similar perturbations in host gene regulation. HBV alters the miRNA-mRNA regulatory network in HCC (98). HPV has a distinct viral gene network signature in HPV-positive head and neck squamous cell carcinoma (99). The complexity of gene regulatory networks and diversity of viral mechanisms for disrupting these networks reveals the challenges of pinpointing a single or primary causal factor.

#### Epigenomic Reprogramming

Epigenetic modifications represent an important mode of adaptive and heritable gene regulation. Persistent viral infection can impact host epigenomes in diverse ways. DNA infection in the nucleus can alter the DNA methylation patterns, frequently resulting in hypermethylation of viral and cellular genes (100). Host genome hypermethylation is detected in HPV and EBV associated carcinomas (100). EBV infection can induce host hypermethylation in a number of experimental models, including non-neoplastic gastric epithelial cells (101), telomerase immortalized keratinocytes (102), and gastric carcinoma derived AGS cells (103). Hypermethylation has been correlated with the inactivation of the dioxygenases TET1 (104) and TET2 (105) that play a role in active demethylation. For EBV, LMP1 and LMP2 can induce DNMT1 expression and subsequent methylation of cellular genes for CDH1 (E-cadherin)(106–108) and tumor suppressors p16 and p21 (109, 110). HPV is also found to alter the host epigenome, including DNA methylation and histone modification patterning (111). Other mechanisms for host epigenetic modification in response to foreign DNA have been described, including modulation of the Sting (112) and Apobec (113) pathways.

Viruses can also induce changes in host chromosome conformation leading to the rewiring of gene regulatory circuits. EBNA-LP, EBNA2, and EBNA3C have been implicated in the reorganization of DNA regulatory loops to form super-enhancers regulating cellular oncogenes, such as c-myc, to drive resting B-lymphocytes into proliferating and immortalized lymphoblastoid cells (91, 114). EBNA2 has been shown to interact cooperatively with several cellular transcription factors, including EBF1 and RBPJ (89), and RUNX1 (115), and this cooperativity may explain some of the capacity to facility new DNA-DNA loop interactions. Episomal viruses may also influence host chromatin and histone modifications through chromosome tethering mechanism. For example, one study found EBV tethering to reinforce heterochromatic H3K9me3 silencing of neuronal genes in EBV positive BL cells (116). Another study found that EBV genome tethering caused transition of heterochromatic H3K9me3 to euchromatic H3K4me3 along with transcriptional activation of cancer-related genes in EBVaGC (117). EBV genomes were also found to transit within open chromosome territories during the switch to reactivation (118). In contrast to viral integrations, episomal tethering may be dynamic over time and provide epigenetic plasticity to both virus and host.

## COMMON THREADS: ONCOVIRAL PERSISTENCE AND PLASTICITY

### Viral Persistence at Tumor Sites

All known human tumor viruses persist, in one form or another, at the site of tumor formation. Tumor viruses can persist as chronic infections (HCV), nuclear episomes (HBV, HPV, MCPyV, EBV, KSHV) or integrated genomes in viral-associated tumor cells (HTLV-1). HCV is an unusual oncovirus in that it does not infect the cancer cell, but its long-term persistence causes inflammation conducive to cancer cell emergence. In contrast, episomal DNA tumor viruses, like EBV, KSHV and HPV have viral-specific programs dedicated to viral genome persistence in a dividing cell that can serve as a clonal outgrowth in cancer (119–121). These viruses encode proteins, such as EBNA1, LANA, and E2, dedicated to binding viral DNA and maintaining the viral genome over generations in proliferating cells. HTLV-1 persists through integration into the host genome as part of its normal life cycle (122). Integration is inherently mutagenic and there is evidence that some, albeit rare integrations are oncogenic.

Chang and Moore proposed that viral cancers arise due to aberrations in the normal productive life cycle, including genetic mutations and integrations that disrupt normal viral gene expression (123). Consistent with this, aberrant integrations are frequently observed for oncoviruses that typically persist as extrachromosomal episomes, such as HBV, HPV, and MCPyV. HBV has been found to integrate in oncogene hot-spots, such as the TERT (telomerase) or KMT2B (MLL4) loci (124), and HPV has been found to integrate at ERBB2 and PTPN13 loci (125). In each case, integration alters normal gene regulation to promote oncogenesis. Integrations can also lead to loss of viral DNA and deregulation of viral oncogenes. HPV integration with loss of viral E2 repressor protein leads to the upregulation of viral oncogenes E6 and E7 in cervical carcinomas (126, 127). In Merkel cell carcinomas, MCPyV frequently integrates as incomplete genomes with deletions in large T and overexpression of small T (18, 128). There is some evidence that viruses can transform host cells without viral genome persistence through heritable changes in the host epigenome or regulome (129). Such “hit and run” mechanisms have been reported but are difficult to demonstrate in naturally occurring human cancers. Thus, most viral cancers are associated with long-term persistence of viral genetic material transmitted for multiple cellular generations.

### Viral and Host Heterogeneity as Oncogenic Drivers

Tumor cell heterogeneity is a central hallmark of cancer (130–134). Viruses can be highly heterogeneous, as well as induce a more heterogeneous phenotype in the host cells. Viral gene programs are inherently unstable with potential shifts from productive to non-productive, or latent to lytic infection cycles with variable gene expression patterns. Viral genomes may be more relaxed than host chromatin and free to move from one chromosome compartment to another. Viral genes may be activated or repressed with greater flexibility, including sporadic bursts of lytic amplification and gene expression. Oncoviruses can also increase variability in host gene expression. Viral infected tumor cells maintain a poorly differentiated state, and may toggle between the lympho-epithelial features of EBV NPC and GC, and the mesenchymal-endothelial features of KSHV infected KS spindle cells (135). This loss of fixed cell identity has been referred to as cellular plasticity. Increasing cellular plasticity provides cancer cells with the advantage of increase variation, or the capacity to adapt more rapidly to changing environmental conditions (such as hypoxia), relocate to new niches (metastasize), evade immune surveillance, and develop drug resistance (136).

Viruses can be highly heterogenous and mutate during the course of infection to increase carcinogenic risk. High and low risk subtypes of HPV may be considered a form of species heterogeneity, while integrations and deletions can be a source of mutational variation (123, 137). Tumor heterogeneity based on viral and host gene expression (including single cell RNAseq), epigenetic modifications, and immune infiltration have been observed for cancers associated with HPV (138), MCPyV (139), HBV (140), HCV (141), and HTLV-1 (142), indicating that such heterogeneity is a general rule for viral and non-viral cancers. Variations in viral gene expression and genome copy number may also account for cancer-risk. For EBV, viral latency types can contribute to genetic heterogeneity and plasticity. EBV can adopt different latency types in different host cells and tumor types. Epigenetic factors are known to regulate the different latency types, including differences in DNA methylation patterning. Among the viral genes with variable expression is the potent oncogene LMP1, involved in constitutive TNF-pathway signaling. LMP1 gene can be expressed at ranges that vary 100 fold among single cells in a population (143). Variations in LMP1 expression in NPC correlated with cellular genes linked to E2F and cellular DNA replication, as well as to changes in NF-kB and JAK/STAT signaling (144). Another variable in viral gene expression is the genome copy number (145). Viral episomes can range from 1 to several 100 copies per cell. Viral genome amplification is closely linked with over-expression of some viral genes, especially the lytic cycle genes of the gammaherpesviruses. Although a complete lytic cycle is rarely detected in viral tumors, abortive lytic cycle gene expression is likely to contribute to viral carcinogenesis. These lytic cycle genes provide numerous potential contributions to viral carcinogenesis, including anti-apoptotic, immunomodulatory, and paracrine activities required for tumorigenesis (146).

### Temporal Heterogeneity and Cancer Cell Evolution

**FIGURE 2 |:**
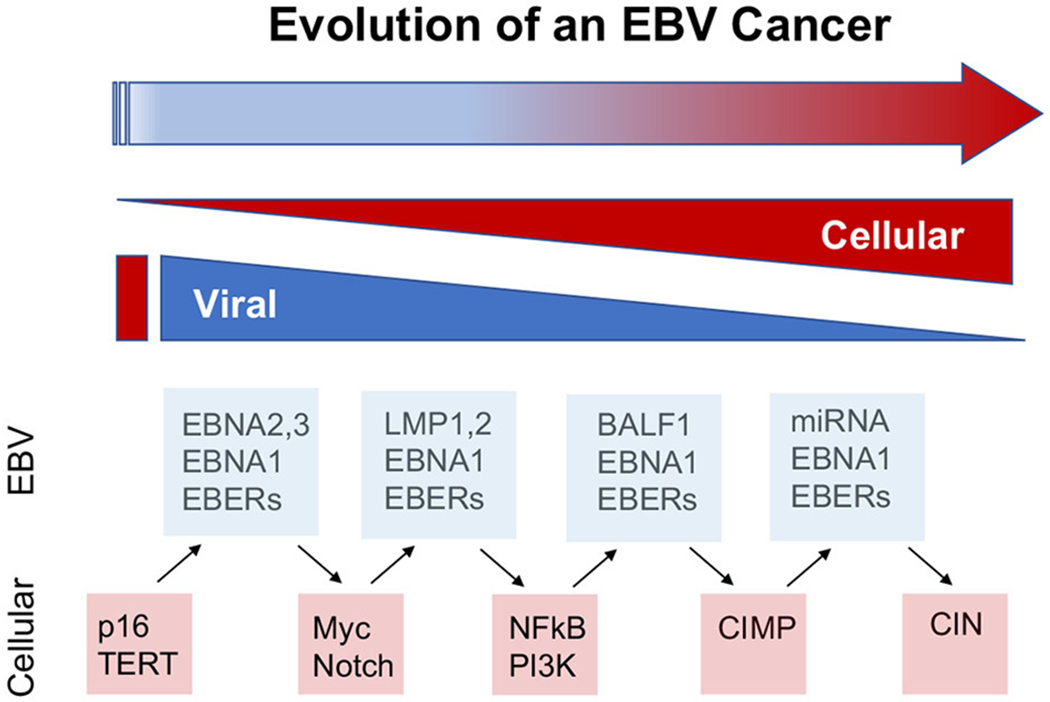
Viral contributions to tumor evolution over time. The viral contributions to carcinogenesis may differ and change depending on the temporal stage of cancer cell evolution. The dynamic properties of viral genomes and infection enable greater plasticity than cellular genomes and gene expression mechanisms. Examples from EBV suggest that viral cancers evolve through changes in viral and cellular gene expression, including the loss of viral oncogenes, such as EBNA2 in BL and LMP1 in NPC, and compensatory oncogenic mutations in cellular genes, such as myc translocations and NFkB activation, at later stages of cancer cell development. In this way, viruses provide lower cost pathways to cellular oncogenesis.

**FIGURE 3 |:**
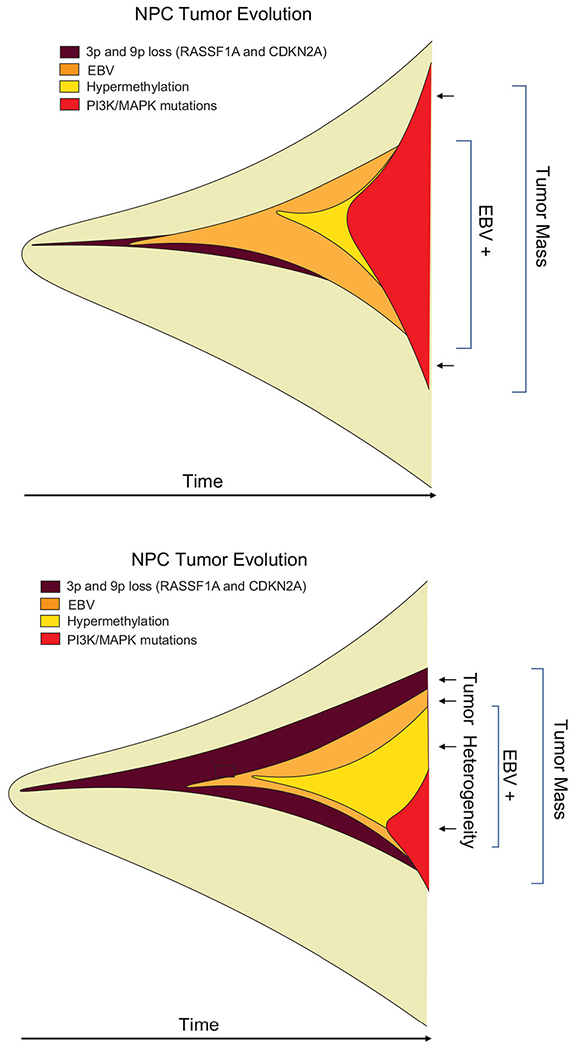
Heterogeneity of viral tumors. Hypothetical “fishplot” of EBV NPC tumors demonstrate highly heterogeneous patterns of viral and host gene expression in the different cells of an emerging tumor (151). Fishplots measure the clonal evolution of cells in a tumor microenvironment over time (152). Such fishplots reflect temporal, historical events in the cancer evolution process, and spatial, topological variations in the tumor microenvironment. Viruses contribute to the adaptability of tumor cells to these rapid changes in microenvironment.

Temporal history of viral infection may also contribute to tumor heterogeneity (Figure 2). Viral cancers are often clonal expansions of a virally infected progenitor cell (147, 148). However, the progenitor cell is likely to have acquired somatic mutations or other aberrations enabling viral transformation. Pre-existing somatic cell mutations appear to be required for the formation of EBV-associated NPC (149). EBV infected nasopharyngeal cells undergo cell cycle arrest, unless infected cells have pre-existing mutations in cell cycle control. Gene loss from 9p21 to 3p21.3 (inactivating RASSF1A and CDKN2A1, respectively), and activation of telomerase, have been found to precede acquisition of EBV infection in the evolution of NPC (150). EBV infection further drives clonal expansion of infected cells, as has been demonstrated in NPC by examining the uniformity of the viral terminal repeat DNA (148). Subsequent genomic hypermethylation follows the infection of EBV, and then activation of NF-kB pathways, loss of MHC I, mutations in PI3K/MAPK, and chromatin remodeling, and subsequently TP53 and RAS, along with other mutations (151) are frequently observed in the course of NPC formation (Figure 3).

Host genetic variation, whether inherited or acquired through somatic mutation, also contribute to the risk of viral cancers. Variations in the HLA locus correlate with risk of viral cancers, suggesting that presentation of viral antigens plays a key role in immune resistance to viral-driven cancers. Genetic analysis of NPC susceptibility revealed risk loci at hTERT, CDKN2A/B, MECOM, and TNFRSF19, all of which have known roles in oncogenic pathways. Other susceptibility pathways have been linked to Notch signaling, magnesium transport (NIPAL1), EBV entry into epithelial cells (ITGB6), modulation of apoptosis (NEDD4L, BCL2L12), cAMP signaling, or DNA repair (MLH1, PRKDC) (153). Inherited mutations in magnesium channel MAGT, as found in XMEN syndrome is associated with defects in NK and T-cell control of EBV infected B-cell (154, 155), and may also contribute to risk of NPC and KSHV associated KS.

Other aberrations also contribute to variations in viral-host interaction. Rare tumors of atypical tissue types, such as EBV leiomyosarcomas, NK-T cell lymphoma, peripheral T-cell lymphoma (156) and pulmonary lympho-epithelium-like carcinoma (LELC) (157) are likely due to aberrant entry of EBV into unnatural host cells. Environmental factors, such as coinfection with HIV or malaria, can alter the immune control of viral infected cells. Thus, host cell type, immune functionality and other environmental factors can impact the course of infection and cancer progression in a temporal-dependent manner.

Stage specific effects on viral oncogenesis are observed for most oncoviruses. HPV has been shown to have different patterns of infection, integration, and gene expression at different stages of viral-associated cancers (158). Similarly, MCPyV T-antigen was found to have stage specific tumor promoting activity in a mouse model treated with defined carcinogens (159). HTLV-1 antigen expression changes in response to T-cell activity, providing evidence for viral adaptation and co-evolution with tumor cell progression (160). HBV X-protein interaction with miRNA production impacts multiple stages of HCC through very different pathways, ranging from cell cycle control at early stages to immune suppression at later stages (161). These multifunctional viral oncoproteins can affect stage specific events in cancer cell evolution, and therefore may adapt their oncogenic activities activities over time.

## SYSTEMS APPROACHES TO VIRAL ONCOLOGY

### Virus-Driven Gene Regulatory Networks and Attractor States

**FIGURE 4 |:**
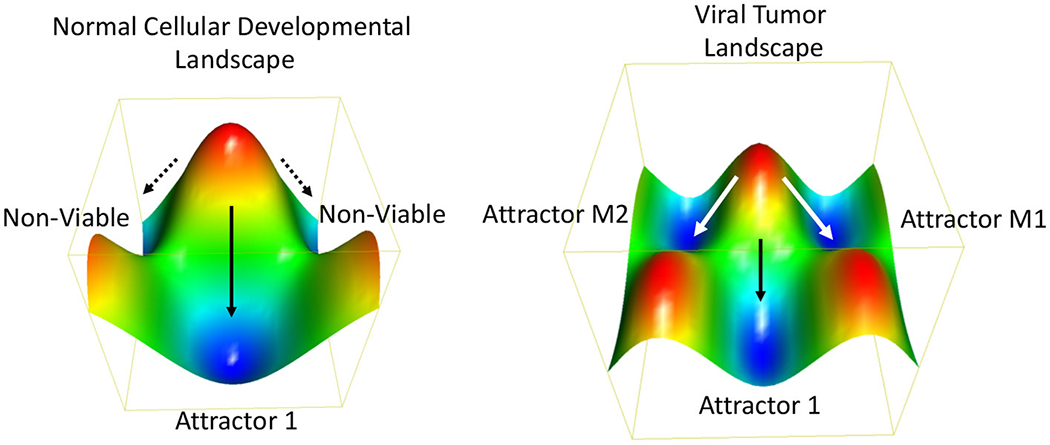
Thermodynamic landscape of viral oncogenesis. A Waddington-like developmental landscape conceptualizes how viruses create alternative gene programs and biochemical pathways that facilitate the transition to an oncogenic state (163). The oncogenic state may be considered in terms of alternative attractor states (e.g., M1 and M2) with favorable thermodynamic properties and increased Darwinian fitness. The Waddington developmental landscape is related to the thermodynamic landscape for chemical reactions. Oncogenic viruses enable new attractors states by providing additional genetic and biochemical flexibility. This viral-borne adaptability may be considered an entropic driver of cancer cell evolution.

Oncogenic viruses increase the number of possible ways a cell can propagate, survive and achieve oncogenic transformation. In terms of Darwinian evolutionary dynamics and population genetics, virus infection increases the phenotypic diversity and fitness of the population. A more heterogeneous population has a fitness advantage by adapting more rapidly to changing and stressed environments. To borrow from systems biology, a stable phenotype requires a stable gene regulatory network (GRN). GRNs are considered a thermodynamic “attractor” state (162) (Figure 4). GRNs are related to the developmental states described by Waddington (164) using an energy landscape and the canalization patterns that separate two or more distinct cell fates or GRNs. It has been proposed that cancer cells converge on a common GRN attractor state akin to the embryonic and unicellular cell states (165). Oncogenic viruses perturb major hubs in GRNs enabling greater plasticity between phenotypic states (136). Experimental validation of this concept has been provided by measuring the intrinsic plasticity of EBV positive Burkitt lymphoma cell lines (162). Viruses can also increase signal noise in a GRN [reviewed in (166)]. Viral genomes may have inherently higher “noise” than their cellular counterparts due, in part, to their relaxed epigenetic regulation, subcellular localizations, and copy number variations. Viral genomes and gene products destabilize GRNs and facilitate the transition from one attractor state to another (167). Thus, we propose that a major feature of oncogenic viruses is their ability to accelerate the rates of cancer cell evolution by increasing the genetic variability and phenotypic plasticity, and inherent cellular adaptability to changing and stressful microenvironments (168, 169).

### Viruses as “Entropic” Drivers of Cancer Evolution

In thermodynamics and statistical mechanics, terms like entropy were developed to explain the behavior of complex systems with excessively large numbers (ensembles) of microstates. In this respect, the term entropy can be used to describe the number of microstates of a complex biological system, such as the gene regulatory interactions in a viral infected tumor cell. While we can not provide a rigorous definition of biological entropy, we do suggest that viruses increase the number of possible microstates available to the host cell, and therefore may be considered a form of “genetic entropy.” In the most simplistic terms, genetic entropy may be the ability to reconfigure the genome and its programmed processes. Viruses reconfigure genomes, gene expression programs, and biochemical pathways. We further suggest that this be considered in terms of Shannon information theory, where viral genomes may be considered a source of signal noise enabling the freedom to find a lower energy state, or alternative GRN, such as the oncogenic state. Viruses can increase signal noise and alternative outputs for cellular developmental programs, and this enables transcriptional plasticity and phenotype heterogeneity associated with tumorigenesis. Efforts to quantify cellular information and thermodynamic entropy may be useful for understanding the emergence of the cancer phenotype. In this regard, virus infection may be considered an entropic driving force for cancer (170).

### Implications for Cancer Therapy

The diversity of oncogenic mechanisms and the plasticity of cancer cells raise enormous challenges in developing precision therapies. For most cancers, early detection provides the best opportunity for effective treatment. Viral cancers have the advantage of having viral-specific targets and biomarkers. For example, early-stage NPC can be predicted from cell-free EBV DNA in plasma (171) and EBV-specific IgA (172) and can be effectively treated with radiation. However, most cancers are discovered at later stages and fail treatment or develop resistance and recurrence. This is largely attributed to tumor heterogeneity and plasticity and the emergence of resistant clonal populations. Oncogenic viruses provide a rich resource for tumor cell heterogeneity and evolutionary diversity. Elimination of persistent oncogenic viruses at early stages is ideal, but not always possible. Reduction of genetic plasticity and modulation of selection pressure may be attractive alternative approaches for treatment of viral cancers. It may also be possible to use evolutionary principles to improve dosing and timing of therapy. Additionally, it may be possible to exploit viral genetic plasticity to eliminate viral cancers. Since excessive genetic variability can be incompatible with life (173), it may be possible to amplify viral-induced plasticity through drug intervention. Treatments that increase gene regulatory noise, such as epigenetic modifiers, could provoke chaotic and lethal gene expression patterns in cancer cells that would be resisted by normal cells.

## CONCLUSIONS

Viruses are thought to be simple biological systems, yet their contributions to cancer can be fiendishly complex. If we return to the question of why some viruses cause cancers but not closely related others, we have only a partial answer. Among the common features, is that of long-term persistence of tumor virus in localized tissue compartments. Another common feature, as highlighted by Chang and Moore, is the frozen accident of the defective virus entering the wrong cell type, or cell with precancerous mutation, or acquired mutation in host or virus that blocks the natural infection and immune clearance. Host and virus genetic variations can be susceptibility factors that enable viral oncogenesis. One additional feature is the plasticity provided by chronic virus infection, and how that effects the survival options for infected tumor cells and tumor fields. Viruses co-opt and perturb numerous cellular pathways implicated in cancer. These perturbations may occur at different times (temporal heterogeneity) and in different subpopulations (spatial heterogeneity) and may be replaced by cellular oncogenic drivers at different stages of tumor evolution (interchangeability). Viral-specific cancer mechanisms may have unique features and provide new insights into cancer biology and genetic plasticity. Despite this complexity, viral cancers may be considered low hanging fruit for cancer therapeutic intervention. Prevention of virus infection and virus-specific inhibitors have been shown to diminish cancer risk, and immune targeting of viral proteins show clinical promise. Deeper understanding of the basic mechanisms driving cancer cell evolution may also be required for more effective intervention.

Precision medicine requires discrete knowledge of the causal factors in disease. Identification of the specific drivers and treatment with selective drugs for each driver pathway is a reasonable, rational, and reductionist approach to cancer therapy. Viral cancers are likely to have different vulnerabilities than their non-viral counterparts, including mechanisms driving cellular plasticity and evolvability. Studying viral cancers may also help us to solve some of the key questions in cancer biology. What are the rate-limiting steps in cancer evolution? What are the most vulnerable nodes of a gene regulatory network for a particular type of cancer and how can we dampen genetic and environmental noise to reduce cancer cell plasticity and evolvability? Can we reverse tissue microenvironment conditions that preferentially select for cancer cell evolution? Ultimately, understanding the evolutionary and thermodynamic driving forces of virus infection and carcinogenesis will provide a more coherent conceptual framework for research and new avenues for therapy.

## GLOSSARY

DREAM: Dimerization partner (DP), Retinoblastoma (RB)-like, E2F and MuvB Complex
EBV: Epstein-Barr Virus
EBVaGC: Epstein-Barr Virus associated Gastric Carcinoma
ER: Endoplasmic reticulum
EV: Extracellular Vessicles
GRN: Gene Regulatory Network
GPCR: G-protein coupled receptor
KSHV: Kaposi’s Sarcoma Associated Herpesvirus
HSV: Herpes Simplex Virus
HTLV-1: Human T-cell Leukemia Virus 1
MCPyV: Merkel Cell Polyomavirus
NK: Natural Killer
HCC: Hepatocellular carcinoma
HDACs: Histone Deacetylase
HLA: Human Leukocyte Antigen
NPC: Nasopharyngeal Carcinoma
NKTCL: Natural Killer-T Cell Lymphoma
NS5A: Non Structural Protein 5A (Influenza)
OXPHOS: Oxidative Phosphorylation
SV40: Simian Virus 40
XMEN: X-linked immuno-deficiency with magnesium defect, Epstein-Barr virus infection, and neoplasia

## Viral Genes—EBV Genes:

BGLF4: Kinase
BALF1: Anti-apoptotic
BART: Non-coding RNAs
BHRF1: Anti-apoptotic
BZLF1: Immediate early b-Zip protein
EBNAs: Epstein-Barr Nuclear Antigens (EBNA-LP, EBNA1, EBNA2, EBNA3A EBNA3B, EBNA3C
LMP1: Latency Membrane Protein 1 (EBV oncogene)
LMP2: Latency Membrane Protein 1 (EBV oncogene)

## KSHV Genes:

LANA: Latency Associated Nuclear Antigen
ORF16: Open Reading Frame 16
vIRFs: Viral Interferon Regulatory Factors

## Polyoma (SV40, MCPyV):

Tag: Tumor Antigen
LT: Large T antigen
ST: Small T antigen

## Adenovirus:

E1A: E1B—Early 1A, 1B

## HPV:

E5: E6, E7—Early 5, 6, 7
HBV: HbX
HTLV-1: Tax
HBZ: Antisense B-zip protein

## Cellular Genes and Proteins:

APH1B: Aph-1 Homolog B, Gamma-Secretase Subunit)
APOBEC: Apolipoprotein B mRNA Editing Catalytic protein
AMPK: Adenosine Monophosphate Kinase
Bcl2: Breakpoint cluster 2 (anti-apoptotic)
Bcl-xL: BCL2 like
Bid: BH3 Interacting Domain Death Agonist
BIRC5: Baculoviral IAP Repeat Containing 5/aka Survivin
Bim: Bcl2 like protein 11 (BCL211)
CDKN2A1: Cyclin-dependent kinase inhibitor 2A (aka p16)
CTLA4: Cytotoxic T-Lymphocyte Associated Protein 4
DNMT: DNA methyltransferase
EP400: E1A Binding Protein P400
GRP: Gastrin Releasing Peptide
HIF1: Hypoxia Inducible Factor 1
IRF: Interferon Regulatory Factor
JAK/STAT: Janus Kinase/Signal Transducer and Activator of Transcription
KMT2B: Lysine Methyl Transferase 2B
NF-kB: Nuclear Factor kB
OCT4: Octamer Binding Factor 4
PD-L1: Programmed death-ligand 1 (CD274)
PI3K: Phosphatidylinositol 4,5-bisphosphate 3-kinase
PTPN: Protein Tyrosine Phosphatase Non-Receptor Type
PU.1: Purine Rich Box Binding factor 1
Puma: p53 upregulated modulator of apoptosis
P16: Tumor suppressor (CDKN1A1)
P53: Tumor suppressor protein 53
gC1qR: Globular heads of the human C1q receptor
MAGT: Magnesium Transporter 1
MAPK: Mitogen-Activated Protein Kinase 1
MLL: Mixed Lymphocytic Leukemia
Myc: Myc Proto-oncogene
MYCL: Myc-like gene
mTOR: Mechanistic Target Of Rapamycin Kinase
Ras: Ras oncogene
RASSF1A: Ras Association Domain Family Member 1
Rb: Retinoblastoma Tumor suppressor gene
RBPJ: Recombination Signal Binding Protein For Immunoglobulin Kappa J Region
RUNX1: Runt-related transcription factor 1
SIGLEC: Sialic Acid Binding Ig Like Lectin 1
SOCS2: Suppressor Of Cytokine Signaling 2
Src: Rous sarcoma virus oncogene
SREBP2: Sterol regulatory element (SRE)-binding protein-2
STING: Stimulator of interferon **genes**
TET: Ten-Eleven Translocation (methylcytosine dioxygenase)
TLR: Toll-Like Receptor
TNF: Tumor Necrosis Factor
TRAFF: TNF receptor (TNFR) associated factor
TRADD: TNFRSF1A Associated Via Death Domain

## References

[1] Parkin DM. The global health burden of infection-associated cancers in the year 2002. Int J Cancer. (2006) 118:3030–44. doi: 10.1002/ijc.21731 16404738

[2] de Martel C, Georges D, Bray F, Ferlay J, Clifford GM. Global burden of cancer attributable to infections in 2018: a worldwide incidence analysis. Lancet Glob Health. (2020) 8:e180–90. doi: 10.1016/S2214-109X(19)30488-7 31862245

[3] Hanahan D, Weinberg RA. The hallmarks of cancer. Cell. (2000) 100:57–70. doi: 10.1016/S0092-8674(00)81683-9 10647931

[4] Hanahan D, Weinberg RA. Hallmarks of cancer: the next generation. Cell. (2011) 144:646–74. doi: 10.1016/j.cell.2011.02.013 21376230

[5] Mesri EA, Feitelson MA, Munger K. Human viral oncogenesis a cancer hallmarks analysis. Cell Host Microbe. (2014) 15:266–82. doi: 10.1016/j.chom.2014.02.011 24629334 PMC3992243

[6] Zur Hausen H Cancers in humans: a lifelong search for contributions of infectious agents, autobiographic notes. Annu Rev Virol. (2019) 6:1–28. doi: 10.1146/annurev-virology-092818-015907 31567062

[7] Pipas JM. DNA tumor viruses and their contributions to molecular biology. J Virol. (2019) 93:e01524–18. doi: 10.1128/JVI.01524-18 30814278 PMC6475781

[8] Lion T Adenovirus persistence, reactivation, clinical management. FEBS Lett. (2019) 593:3571–82. doi: 10.1002/1873-3468.13576 31411731

[9] Munger K, Scheffner M, Huibregtse JM, Howley PM. Interactions of HPV E6 and E7 oncoproteins with tumour suppressor gene products. Cancer Surv. (1992) 12:197–217 1322242

[10] Rashid NN, Rothan HA, Yusoff MS. The association of mammalian DREAM complex and HPV16 E7 proteins. Am J Cancer Res. (2015) 5:3525–33. 26885443 PMC4731628

[11] Hatterschide J, Bohidar AE, Grace M, Nulton TJ, Kim HW, Windle B, . PTPN14 degradation by high-risk human papillomavirus E7 limits keratinocyte differentiation and contributes to HPV-mediated oncogenesis. Proc Natl Acad Sci U S A. (2019) 116:7033–42. doi: 10.1073/pnas.1819534116 30894485 PMC6452706

[12] Longworth MS, Laimins LA. The binding of histone deacetylases and the integrity of zinc finger-like motifs of the E7 protein are essential for the life cycle of human papillomavirus type 31. J Virol. (2004) 78:3533–41. doi: 10.1128/JVI.78.7.3533-3541.2004 15016876 PMC371089

[13] Yang S, Chen T, Huang L, Xu S, Cao Z, Zhang S, . High-risk human papillomavirus E7 maintains stemness *via* APH1B in cervical cancer stem-cell like cells. Cancer Manag Res. (2019) 11:9541–52. doi: 10.2147/CMAR.S194239 31814758 PMC6858839

[14] Panayiotou T, Michael S, Zaravinos A, Demirag E, Achilleos C, Strati K. Human papillomavirus E7 binds Oct4 and regulates its activity in HPV-associated cervical cancers. PLoS Pathog. (2020) 16:e1008468. doi: 10.1371/journal.ppat.1008468 32298395 PMC7228134

[15] Westrich JA, Warren CJ, Klausner MJ, Guo K, Liu CW, Santiago ML, . Human papillomavirus 16 E7 stabilizes APOBEC3A protein by inhibiting cullin 2-dependent protein degradation. J Virol. (2018) 92:e01318–17. doi: 10.1128/JVI.01318-17 29367246 PMC5972886

[16] Liu X, Roberts J, Dakic A, Zhang Y, Schlegel R, HPV. E7 contributes to the telomerase activity of immortalized and tumorigenic cells and augments E6-induced hTERT promoter function. Virology. (2008) 375:611–23. doi: 10.1016/j.virol.2008.02.025 18367227 PMC2716003

[17] Katzenellenbogen RA. Activation of telomerase by HPVs. Virus Res. (2017) 231:50–5. doi: 10.1016/j.virusres.2016.11.003 27863966

[18] Park DE, Cheng J, McGrath JP, Lim MY, Cushman C, Swanson SK, . Merkel cell polyomavirus activates LSD1-mediated blockade of noncanonical BAF to regulate transformation and tumorigenesis. Nat Cell Biol. (2020) 22:603–15. doi: 10.1038/s41556-020-0503-2 32284543 PMC7336275

[19] Shuda M, Kwun HJ, Feng H, Chang Y, Moore PS. Human Merkel cell polyomavirus small T antigen is an oncoprotein targeting the 4E-BP1 translation regulator. J Clin Invest. (2011) 121:3623–34. doi: 10.1172/JCI46323 21841310 PMC3163959

[20] Kwun HJ, Shuda M, Camacho CJ, Gamper AM, Thant M, Chang Y, Restricted protein phosphatase 2A targeting by Merkel cell polyomavirus small T antigen. J Virol. (2015) 89:4191–200. doi: 10.1128/JVI.00157-15 25631078 PMC4442354

[21] Nwogu N, Ortiz LE, Kwun HJ. Surface charge of Merkel cell polyomavirus small T antigen determines cell transformation through allosteric FBW7 WD40 domain targeting. Oncogenesis. (2020) 9:53. doi: 10.1038/s41389-020-0235-y 32427880 PMC7237485

[22] Zhao J, Jia Y, Shen S, Kim J, Wang X, Lee E, Merkel cell polyomavirus small T antigen activates noncanonical NFkappaB signaling to promote tumorigenesis. Mol Cancer Res. (2020) 18:1623–37. doi: 10.1158/1541-7786.MCR-20-0587 32753470 PMC7641980

[23] Tsang SH, Wang R, Nakamaru-Ogiso E, Knight SA, Buck CB, You J. The oncogenic small tumor antigen of Merkel cell polyomavirus is an iron-sulfur cluster protein that enhances viral DNA replication. J Virol. (2016) 90:1544–56. doi: 10.1128/JVI.02121-15 26608318 PMC4719616

[24] Thorley-Lawson DA. Epstein-Barr virus: exploiting the immune system. Nat Rev Immunol. (2001) 1:75–82. doi: 10.1038/35095584 11905817

[25] Thorley-Lawson DA. EBV persistence-introducing the virus. Curr Top Microbiol Immunol. (2015) 390:151–209. doi: 10.1007/978-3-319-22822-8_8 26424647 PMC5125397

[26] Wang LW, Jiang S, Gewurz BE. Epstein-Barr virus LMP1-Mediated oncogenicity. J Virol. (2017) 91:e01718–16. doi: 10.1128/JVI.01718-16 28835489 PMC5640852

[27] Rider MA, Cheerathodi MR, Hurwitz SN, Nkosi D, Howell LA, Tremblay DC, The interactome of EBV LMP1 evaluated by proximity-based BioID approach. Virology. (2018) 516:55–70. doi: 10.1016/j.virol.2017.12.033 29329079 PMC5826876

[28] Longnecker R Epstein-Barr virus latency: LMP2, a regulator or means for Epstein-Barr virus persistence? Adv Cancer Res. (2000) 79:175–200. doi: 10.1016/S0065-230X(00)79006-3 10818681

[29] Farrell PJ. Epstein-Barr virus and cancer. Annu Rev Pathol. (2019) 14:29–53. doi: 10.1146/annurev-pathmechdis-012418-013023 30125149

[30] Pich D, Mrozek-Gorska P, Bouvet M, Sugimoto A, Akidil E, Grundhoff A, First days in the life of naive human B lymphocytes infected with Epstein-Barr sirus. mBio. (2019) 10. doi: 10.1128/mBio.01723-19 PMC6751056 31530670

[31] Ponnusamy R, Khatri R, Correia PB, Wood CD, Mancini EJ, Farrell PJ, Increased association between Epstein-Barr virus EBNA2 from type 2 strains and the transcriptional repressor BS69 restricts EBNA2 activity. PLoS Pathog. (2019) 15:e1007458. doi: 10.1371/journal.ppat.1007458 31283782 PMC6638984

[32] Cesarman E, Damania B, Krown SE, Martin J, Bower M, Whitby D. Kaposi sarcoma. Nat Rev Dis Primers. (2019) 5:9. doi: 10.1038/s41572-019-0060-9 30705286 PMC6685213

[33] Lee N, Moss WN, Yario TA, Steitz JA. EBV noncoding RNA binds nascent RNA to drive host PAX5 to viral DNA. Cell. (2015) 160:607–18. doi: 10.1016/j.cell.2015.01.015 25662012 PMC4329084

[34] Houmani JL, Davis CI, Ruf IK. Growth-promoting properties of Epstein-Barr virus EBER-1 RNA correlate with ribosomal protein L22 binding. J Virol. (2009) 83:9844–53. doi: 10.1128/JVI.01014-09 19640998 PMC2747990

[35] Li Z, Duan Y, Cheng S, Chen Y, Hu Y, Zhang L, EBV-encoded RNA *via* TLR3 induces inflammation in nasopharyngeal carcinoma. Oncotarget. (2015) 6:24291–303. doi: 10.18632/oncotarget.4552 26172457 PMC4695186

[36] Baglio SR, van Eijndhoven MA, Koppers-Lalic D, Berenguer J, Lougheed SM, Gibbs S, Sensing of latent EBV infection through exosomal transfer of 5’pppRNA. Proc Natl Acad Sci U S A. (2016) 113:E587–596. doi: 10.1073/pnas.1518130113 26768848 PMC4747727

[37] Hassani A, Khan G. Epstein-Barr virus and miRNAs: partners in crime in the pathogenesis of multiple sclerosis? Front Immunol. (2019) 10:695. doi: 10.3389/fimmu.2019.00695 31001286 PMC6456696

[38] Hussein HAM, Alfhili MA, Pakala P, Simon S, Hussain J, McCubrey JA, miRNAs and their roles in KSHV pathogenesis. Virus Res. (2019) 266:15–24. doi: 10.1016/j.virusres.2019.03.024s 30951791

[39] Shinozaki-Ushiku A, Kunita A, Fukayama M. Update on Epstein-Barr virus and gastric cancer (review). Int J Oncol. (2015) 46:1421–34. doi: 10.3892/ijo.2015.2856 25633561

[40] Zhang J, Li X, Hu J, Cao P, Yan Q, Zhang S, Long noncoding RNAs involvement in Epstein-Barr virus infection and tumorigenesis. Virol J. (2020) 17:51. doi: 10.1186/s12985-020-01308-y 32272952 PMC7146903

[41] Liang C, Oh BH, Jung JU. Novel functions of viral anti-apoptotic factors. Nat Rev Microbiol. (2015) 13:7–12. doi: 10.1038/nrmicro3369 25363821 PMC4420620

[42] Banerjee S, Uppal T, Strahan R, Dabral P, Verma SC. The modulation of apoptotic pathways by gammaherpesviruses. Front Microbiol. (2016) 7:585. doi: 10.3389/fmicb.2016.00585 27199919 PMC4847483

[43] Kvansakul M Viral infection and apoptosis. Viruses. (2017) 9:356. doi: 10.3390/v9120356 PMC5744131 29168732

[44] Altmann M, Hammerschmidt W. Epstein-Barr virus provides a new paradigm: a requirement for the immediate inhibition of apoptosis. PLoS Biol. (2005) 3:e404. doi: 10.1371/journal.pbio.0030404 16277553 PMC1283332

[45] Fitzsimmons L, Kelly GL. EBV and apoptosis: the viral master regulator of cell fate? Viruses. (2017) 9:339. doi: 10.3390/v9110339 PMC5707546 29137176

[46] Marquitz AR, Mathur A, Nam CS, Raab-Traub N. The Epstein-Barr virus BART microRNAs target the pro-apoptotic protein bim. Virology. (2011) 412:392–400. doi: 10.1016/j.virol.2011.01.028 21333317 PMC3340891

[47] Choy EY, Siu KL, Kok KH, Lung RW, Tsang CM, To KF, An Epstein-Barr virus-encoded microRNA targets PUMA to promote host cell survival. J Exp Med. (2008) 205:2551–60. doi: 10.1084/jem.20072581 18838543 PMC2571930

[48] Gallo A, Lampe M, Gunther T, Brune W. The viral Bcl-2 homologs of Kaposi’s sarcoma-associated herpesvirus and rhesus rhadinovirus share an essential role for viral replication. J Virol. (2017) 91:e01875–16. doi: 10.1128/JVI.01875-16 28053098 PMC5331788

[49] Arora R, Shuda M, Guastafierro A, Feng H, Toptan T, Tolstov Y, Survivin is a therapeutic target in Merkel cell carcinoma. Sci Transl Med. (2012) 4:133ra156. doi: 10.1126/scitranslmed.3003713 PMC3726222 22572880

[50] Zhang TY, Chen HY, Cao JL, Xiong HL, Mo XB Li TL, Structural and functional analyses of hepatitis B virus X protein BH3-like domain and Bcl-xL interaction. Nat Commun. (2019) 10:3192. doi: 10.1038/s41467-019-11173-1 31324803 PMC6642116

[51] Jiang X, Kanda T, Wu S, Nakamoto S, Wakita T, Shirasawa H, Hepatitis C virus nonstructural protein 5A inhibits thapsigargin-induced apoptosis. PLoS ONE. (2014) 9:e113499. doi: 10.1371/journal.pone.0113499 25409163 PMC4237446

[52] Muhleisen A, Giaisi M, Kohler R, Krammer PH, Li-Weber M. Tax contributes apoptosis resistance to HTLV-1-infected T cells *via* suppression of Bid and Bim expression. Cell Death Dis. (2014) 5:e1575. doi: 10.1038/cddis.2014.536 25522269 PMC4649845

[53] Pavlova NN, Thompson CB. The emerging hallmarks of cancer metabolism. Cell Metab. (2016) 23:27–47. doi: 10.1016/j.cmet.2015.12.006 26771115 PMC4715268

[54] Purdy JG, Luftig MA. Reprogramming of cellular metabolic pathways by human oncogenic viruses. Curr Opin Virol. (2019) 39:60–9. doi: 10.1016/j.coviro.2019.11.002 31766001 PMC6986357

[55] Fan R, Hou WJ, Zhao YJ, Liu SL, Qiu XS, Wang EH, Overexpression of HPV16 E6/E7 mediated HIF-1alpha upregulation of GLUT1 expression in lung cancer cells. Tumour Biol. (2016) 37:4655–63. doi: 10.1007/s13277-015-4221-5 26508030

[56] Nakamura M, Bodily JM, Beglin M, Kyo S, Inoue M, Laimins LA. Hypoxia-specific stabilization of HIF-1alpha by human papillomaviruses. Virology. (2009) 387:442–8. doi: 10.1016/j.virol.2009.02.036 19321184 PMC2674135

[57] Cuninghame S, Jackson R, Lees SJ, Zehbe I. Two common variants of human papillomavirus type 16 E6 differentially deregulate sugar metabolism and hypoxia signalling in permissive human keratinocytes. J Gen Virol. (2017) 98:2310–9. doi: 10.1099/jgv.0.000905 28857035

[58] Zwerschke W, Mazurek S, Massimi P, Banks L, Eigenbrodt E, Jansen-Durr P. Modulation of type M2 pyruvate kinase activity by the human papillomavirus type 16 E7 oncoprotein. Proc Natl Acad Sci U S A. (1999) 96:1291–6. doi: 10.1073/pnas.96.4.1291 9990017 PMC15456

[59] Berrios C, Padi M, Keibler MA, Park DE, Molla V, Cheng J, Merkel cell polyomavirus small T antigen promotes pro-glycolytic metabolic perturbations required for transformation. PLoS Pathog. (2016) 12:e1006020. doi: 10.1371/journal.ppat.1006020 27880818 PMC5120958

[60] Hafez AY, Messinger JE, McFadden K, Fenyofalvi G, Shepard CN, Lenzi GM, Limited nucleotide pools restrict Epstein-Barr virus-mediated B-cell immortalization. Oncogenesis. (2017) 6:e349. doi: 10.1038/oncsis.2017.46 28604764 PMC5519195

[61] Wang LW, Shen H, Nobre L, Ersing I, Paulo JA, Trudeau S, Epstein-Barr-virus-induced one-carbon metabolism drives B cell transformation. Cell Metab. (2019) 30:539–55.e511. doi: 10.1016/j.cmet.2019.06.003 31257153 PMC6720460

[62] Wang LW, Wang Z, Ersing I, Nobre L, Guo R, Jiang S, Epstein-Barr virus subverts mevalonate and fatty acid pathways to promote infected B-cell proliferation and survival. PLoS Pathog. (2019) 15:e1008030. doi: 10.1371/journal.ppat.1008030 31518366 PMC6760809

[63] Yogev O, Lagos D, Enver T, Boshoff C. Kaposi’s sarcoma herpesvirus microRNAs induce metabolic transformation of infected cells. PLoS Pathog. (2014) 10:e1004400. doi: 10.1371/journal.ppat.1004400 25255370 PMC4177984

[64] Bertout JA, Patel SA, Simon MC. The impact of O_2_ availability on human cancer. Nat Rev Cancer. (2008) 8:967–75. doi: 10.1038/nrc2540 18987634 PMC3140692

[65] Hoppe-Seyler K, Mandl J, Adrian S, Kuhn BJ, Hoppe-Seyler F. Virus/Host cell crosstalk in hypoxic HPV-Positive cancer cells. Viruses. (2017) 9:174. doi: 10.3390/v9070174 PMC5537666 28678198

[66] Carroll PA, Kenerson HL, Yeung RS, Lagunoff M. Latent Kaposi’s sarcoma-associated herpesvirus infection of endothelial cells activates hypoxia-induced factors. J Virol. (2006) 80:10802–12. doi: 10.1128/JVI.00673-06 16956952 PMC1641760

[67] Haque M, Wang V, Davis DA, Zheng ZM, Yarchoan R. Genetic organization and hypoxic activation of the Kaposi’s sarcoma-associated herpesvirus ORF34-37 gene cluster. J Virol. (2006) 80:7037–51. doi: 10.1128/JVI.00553-06 16809309 PMC1489055

[68] Sodhi A, Montaner S, Patel V, Zohar M, Bais C, Mesri EA, The Kaposi’s sarcoma-associated herpes virus G protein-coupled receptor up-regulates vascular endothelial growth factor expression and secretion through mitogen-activated protein kinase and p38 pathways acting on hypoxia-inducible factor 1alpha. Cancer Res. (2000) 60:4873–80. 10987301

[69] Giffin L, Yan F, Ben Major M, Damania B. Modulation of Kaposi’s sarcoma-associated herpesvirus interleukin-6 function by hypoxia-upregulated protein 1. J Virol (2014) 88:9429–41. doi: 10.1128/JVI.00511-14 24920810 PMC4136275

[70] Kumar V, Gabrilovich DI. Hypoxia-inducible factors in regulation of immune responses in tumour microenvironment. Immunology. (2014) 143:512–9. doi: 10.1111/imm.12380 25196648 PMC4253499

[71] Magalhaes I, Yogev O, Mattsson J, Schurich A. The metabolic profile of tumor and virally infected cells shapes their microenvironment counteracting T cell immunity. Front Immunol. (2019) 10:2309. doi: 10.3389/fimmu.2019.02309 31636636 PMC6788393

[72] Chang CH, Qiu J, O’Sullivan D, Buck MD, Noguchi T, Curtis JD, Metabolic competition in the tumor microenvironment is a driver of cancer progression. Cell. (2015) 162:1229–41. doi: 10.1016/j.cell.2015.08.016 26321679 PMC4864363

[73] Bentz GL, Liu R, Hahn AM, Shackelford J, Pagano JS. Epstein-Barr virus BRLF1 inhibits transcription of IRF3 and IRF7 and suppresses induction of interferon-beta. Virology. (2010) 402:121–8. doi: 10.1016/j.virol.2010.03.014 20381110 PMC2871977

[74] Morrison TE, Mauser A, Wong A, Ting JP, Kenney SC. Inhibition of IFN-gamma signaling by an Epstein-Barr virus immediate-early protein. Immunity. (2001) 15:787–99. doi: 10.1016/S1074-7613(01)00226-6 11728340

[75] Morrison TE, Mauser A, Klingelhutz A, Kenney SC. Epstein-Barr virus immediate-early protein BZLF1 inhibits tumor necrosis factor alpha-induced signaling and apoptosis by downregulating tumor necrosis factor receptor 1. J Virol. (2004) 78:544–9. doi: 10.1128/JVI.78.1.544-549.2004 14671137 PMC303403

[76] Michaud F, Coulombe F, Gaudreault E, Paquet-Bouchard C, Rola-Pleszczynski M, Gosselin J. Epstein-Barr virus interferes with the amplification of IFNalpha secretion by activating suppressor of cytokine signaling 3 in primary human monocytes. PLoS ONE. (2010) 5:e11908. doi: 10.1371/journal.pone.0011908 20689596 PMC2912847

[77] van Gent M, Griffin BD, Berkhoff EG, van Leeuwen D, Boer IG, Buisson M, EBV lytic-phase protein BGLF5 contributes to TLR9 downregulation during productive infection. J Immunol. (2011) 186:1694–702. doi: 10.4049/jimmunol.0903120 21191071

[78] McNamara RP, Chugh PE, Bailey A, Costantini LM, Ma Z, Bigi R, Extracellular vesicles from Kaposi Sarcoma-associated herpesvirus lymphoma induce long-term endothelial cell reprogramming. PLoS Pathog. (2019) 15:e1007536. doi: 10.1371/journal.ppat.1007536 30716130 PMC6361468

[79] Hurwitz SN, Nkosi D, Conlon MM, York SB, Liu X, Tremblay DC, CD63 regulates Epstein-Barrvirus LMP1 exosomal packaging, enhancement of vesicle production, and noncanonical NF-kappaB signaling. J Virol. (2017) 91:e02251–16. doi: 10.1128/JVI.02251-16 27974566 PMC5309960

[80] Yogev O, Henderson S, Hayes MJ, Marelli SS, Ofir-Birin Y, Regev-Rudzki N, Herpesviruses shape tumour microenvironment through exosomal transfer of viral microRNAs. PLoS Pathog. (2017) 13:e1006524. doi: 10.1371/journal.ppat.1006524 28837697 PMC5570218

[81] Mrizak D, Martin N, Barjon C, Jimenez-Pailhes AS, Mustapha R, Niki T, Effect of nasopharyngeal carcinoma-derived exosomes on human regulatory T cells. J Natl Cancer Inst. (2015) 107:363. doi: 10.1093/jnci/dju363 25505237

[82] Cao S, Wylie KM, Wyczalkowski MA, Karpova A, Ley J, Sun S, Dynamic host immune response in virus-associated cancers. Commun Biol. (2019) 2:109. doi: 10.1038/s42003-019-0352-3 30911684 PMC6430765

[83] Anastasiadou E, Stroopinsky D, Alimperti S, Jiao AL, Pyzer AR, Cippitelli C, Epstein-Barr virus-encoded EBNA2 alters immune checkpoint PD-L1 expression by downregulating miR-34a in B-cell lymphomas. Leukemia. (2019) 33:132–47. doi: 10.1038/s41375-018-0178-x 29946193 PMC6327052

[84] Fang W, Zhang J, Hong S, Zhan J, Chen N, Qin T, EBV-driven LMP1 and IFN-gamma up-regulate PD-L1 in nasopharyngeal carcinoma: implications for oncotargeted therapy. Oncotarget. (2014) 5:12189–202. doi: 10.18632/oncotarget.2608 25361008 PMC4322961

[85] Bi XW, Wang H, Zhang WW, Wang JH, Liu WJ, Xia ZJ, PD-L1 is upregulated by EBV-driven LMP1 through NF-kappaB pathway and correlates with poor prognosis in natural killer/T-cell lymphoma. J Hematol Oncol. (2016) 9:109. doi: 10.1186/s13045-016-0341-7 27737703 PMC5064887

[86] Miyauchi S, Sanders PD, Guram K, Kim SS, Paolini F, Venuti A, HPV16 E5 mediates resistance to PD-L1 blockade and can be targeted with rimantadine in head and neck cancer. Cancer Res. (2020) 80:732–46. doi: 10.1158/0008-5472.CAN-19-1771 31848196 PMC7024675

[87] Tsai TY, Huang MT, Sung PS, Peng CY, Tao MH, Yang HI, SIGLEC-3 (CD33) serves as an immune checkpoint receptor for HBV infection. J Clin Invest. (2021) 131:e141965. doi: 10.1172/JCI141965 PMC8159688 34060491

[88] Osuch S, Metzner KJ, Caraballo Cortes. K. Reversal of T cell exhaustion in chronic HCV infection. Viruses. (2020) 12:799. doi: 10.3390/v12080799 PMC7472290 32722372

[89] Lu F, Chen HS, Kossenkov AV, DeWispeleare K, Won KJ, Lieberman PM. EBNA2 drives formation of new chromosome binding sites and target genes for B-Cell Master regulatory transcription factors RBP-jkappa and EBF1. PLoS Pathog. (2016) 12:e1005339. doi: 10.1371/journal.ppat.1005339 26752713 PMC4709166

[90] Liang J, Zhou H, Gerdt C, Tan M, Colson T, Kaye KM, Epstein-Barr virus super-enhancer eRNAs are essential for MYC oncogene expression and lymphoblast proliferation. Proc Natl Acad Sci U S A. (2016) 113:14121–6 doi: 10.1073/pnas.1616697113 27864512 PMC5150416

[91] Jiang S, Zhou H, Liang J, Gerdt C, Wang C, Ke L, The Epstein-Barr virus Regulome in Lymphoblastoid Cells. Cell Host Microbe. (2017) 22:561–73.e564. doi: 10.1016/j.chom.2017.09.001 29024646 PMC5662195

[92] Skalska L, White RE, Franz M, Ruhmann M, Allday MJ. Epigenetic repression of p16(INK4A) by latent Epstein-Barr virus requires the interaction of EBNA3A and EBNA3C with CtBP. PLoS Pathog. (2010) 6:e1000951. doi: 10.1371/journal.ppat.1000951 20548956 PMC2883600

[93] Paschos K, Parker GA, Watanatanasup E, White RE, Allday MJ. BIM promoter directly targeted by EBNA3C in polycomb-mediated repression by EBV. Nucleic Acids Res. (2012) 40:7233–46. doi: 10.1093/nar/gks391 22584624 PMC3424555

[94] Juillard F, Tan M, Li S, Kaye KM. Kaposi’s sarcoma herpesvirus genome persistence. Front Microbiol. (2016) 7:1149. doi: 10.3389/fmicb.2016.01149 27570517 PMC4982378

[95] Ueda K KSHV genome replication and maintenance in latency. Adv Exp Med Biol. (2018) 1045:299–320. doi: 10.1007/978-981-10-7230-7_14 29896673

[96] Brulois K, Jung JU. Interplay between Kaposi’s sarcoma-associated herpesvirus and the innate immune system. Cytokine Growth Factor Rev. (2014) 25:597–609. doi: 10.1016/j.cytogfr.2014.06.001 25037686 PMC4252609

[97] Giudice A, D’Arena G, Crispo A, Tecce MF, Nocerino F, Grimaldi M, Role of viral miRNAs and epigenetic modifications in Epstein-Barr virus-associated gastric carcinogenesis. Oxid Med Cell Longev. (2016) 2016:6021934. doi: 10.1155/2016/6021934 26977250 PMC4764750

[98] Lou W, Liu J, Ding B, Chen D, Xu L, Ding J, Identification of potential miRNA-mRNA regulatory network contributing to pathogenesis of HBV-related HCC. J Transl Med. (2019) 17:7. doi: 10.1186/s12967-018-1761-7 30602391 PMC6317219

[99] Zhao Q, Zhang Y, Zhang X, Sun Y, Lin Z. Mining of gene modules and identification of key genes in head and neck squamous cell carcinoma based on gene co-expression network analysis. Medicine. (2020) 99:e22655. doi: 10.1097/MD.0000000000022655 33285674 PMC7717835

[100] Kuss-Duerkop SK, Westrich JA, Pyeon D. DNA tumor virus regulation of host DNA methylation and its implications for immune evasion and oncogenesis. Viruses. (2018) 10:82. doi: 10.3390/v10020082 PMC5850389 29438328

[101] Matsusaka K, Funata S, Fukuyo M, Seto Y, Aburatani H, Fukayama M, Epstein-Barr virus infection induces genome-wide *de novo* DNA methylation in non-neoplastic gastric epithelial cells. J Pathol. (2017) 242:391–9. doi: 10.1002/path.4909 28418084

[102] Birdwell CE, Queen KJ, Kilgore PC, Rollyson P, Trutschl M, Cvek U, Genome-wide DNA methylation as an epigenetic consequence of Epstein-Barr virus infection of immortalized keratinocytes. J Virol. (2014) 88:11442–58. doi: 10.1128/JVI.00972-14 25056883 PMC4178815

[103] Kaneda A, Matsusaka K, Aburatani H, Fukayama M. Epstein-Barr virus infection as an epigenetic driver of tumorigenesis. Cancer Res. (2012) 72:3445–50. doi: 10.1158/0008-5472.CAN-11-3919 22761333

[104] Li L, Li C, Mao H, Du Z, Chan WY, Murray P, Epigenetic inactivation of the CpG demethylase TET1 as a DNA methylation feedback loop in human cancers. Sci Rep. (2016) 6:26591. doi: 10.1038/srep34435 27225590 PMC4880909

[105] Namba-Fukuyo H, Funata S, Matsusaka K, Fukuyo M, Rahmutulla B, Mano Y, TET2 functions as a resistance factor against DNA methylation acquisition during Epstein-Barr virus infection. Oncotarget. (2016) 7:81512–26. doi: 10.18632/oncotarget.13130 27829228 PMC5348409

[106] Tsai CN, Tsai CL, Tse KP, Chang HY, Chang YS. The Epstein-Barr virus oncogene product, latent membrane protein 1, induces the downregulation of E-cadherin gene expression *via* activation of DNA methyltransferases. Proc Natl Acad Sci U S A. (2002) 99:10084–9. doi: 10.1073/pnas.152059399 12110730 PMC126628

[107] Tsai CL Li HP, Lu YJ, Hsueh C, Liang Y, Chen CL, Activation of DNA methyltransferase 1 by EBV LMP1 Involves c-Jun NH(2)-terminal kinase signaling. Cancer Res. (2006) 66:11668–76. doi: 10.1158/0008-5472.CAN-06-2194 17178861

[108] Hino R, Uozaki H, Murakami N, Ushiku T, Shinozaki A, Ishikawa S, Activation of DNA methyltransferase 1 by EBV latent membrane protein 2A leads to promoter hypermethylation of PTEN gene in gastric carcinoma. Cancer Res. (2009) 69:2766–74. doi: 10.1158/0008-5472.CAN-08-3070 19339266

[109] Chong JM, Sakuma K, Sudo M, Ushiku T, Uozaki H, Shibahara J, Global and non-random CpG-island methylation in gastric carcinoma associated with Epstein-Barr virus. Cancer Sci. (2003) 94:76–80. doi: 10.1111/j.1349-7006.2003.tb01355.x 12708478 PMC11160188

[110] Okabe A, Funata S, Matsusaka K, Namba H, Fukuyo M, Rahmutulla B, Regulation of tumour related genes by dynamic epigenetic alteration at enhancer regions in gastric epithelial cells infected by Epstein-Barr virus. Sci Rep. (2017) 7:7924. doi: 10.1038/s41598-017-08370-7 28801683 PMC5554293

[111] Soto D, Song C, McLaughlin-Drubin ME. Epigenetic alterations in human papillomavirus-associated cancers. Viruses. (2017) 9:248. doi: 10.3390/v9090248 PMC5618014 28862667

[112] Knipe DM. Nuclear sensing of viral DNA, epigenetic regulation of herpes simplex virus infection, innate immunity. Virology. (2015) 479–80:153–9. doi: 10.1016/j.virol.2015.02.009 PMC4424148 25742715

[113] Knisbacher BA, Gerber D, Levanon EY, DNA editing by APOBECs: a genomic preserver and transformer. Trends Genet. (2016) 32:16–28. doi: 10.1016/j.tig.2015.10.005 26608778

[114] Zhou H, Schmidt SC, Jiang S, Willox B, Bernhardt K, Liang J, Epstein-Barr virus oncoprotein super-enhancers control B cell growth. Cell Host Microbe. (2015) 17:205–16. doi: 10.1016/j.chom.2014.12.013 25639793 PMC4539236

[115] Gunnell A, Webb HM, Wood CD, McClellan MJ, Wichaidit B, Kempkes B, RUNX super-enhancer control through the Notch pathway by Epstein-Barr virus transcription factors regulates B cell growth. Nucleic Acids Res. (2016) 44:4636–50. doi: 10.1093/nar/gkw085 26883634 PMC4889917

[116] Kim KD, Tanizawa H, De Leo A, Vladimirova O, Kossenkov A, Lu F, Epigenetic specifications of host chromosome docking sites for latent Epstein-Barr virus. Nat Commun. (2020) 11:877. doi: 10.1038/s41467-019-14152-8 32054837 PMC7018943

[117] Okabe A, Huang KK, Matsusaka K, Fukuyo M, Xing M, Ong X, Cross-species chromatin interactions drive transcriptional rewiring in Epstein-Barr virus-positive gastric adenocarcinoma. Nat Genet. (2020) 52:919–30. doi: 10.1038/s41588-020-0665-7 32719515

[118] Moquin SA, Thomas S, Whalen S, Warburton A, Fernandez SG, McBride AA, The Epstein-Barr virus episome maneuvers between nuclear chromatin compartments during reactivation. J Virol. (2018) 92. doi: 10.1128/JVI.01413-17 PMC5774889 29142137

[119] Lieberman PM. Keeping it quiet: chromatin control of gammaherpesvirus latency. Nat Rev Microbiol. (2013) 11:863–75. doi: 10.1038/nrmicro3135 24192651 PMC4544771

[120] Lieberman PM. Epigenetics and genetics of viral latency. Cell Host Microbe. (2016) 19:619–28. doi: 10.1016/j.chom.2016.04.008 27173930 PMC5166714

[121] De Leo A, Calderon A, Lieberman PM. Control of viral latency by episome maintenance proteins. Trends Microbiol. (2020) 28:150–62. doi: 10.1016/j.tim.2019.09.002 31624007 PMC6980450

[122] Watanabe T Adult T-cell leukemia: molecular basis for clonal expansion and transformation of HTLV-1-infected T cells. Blood. (2017) 129:1071–81. doi: 10.1182/blood-2016-09-692574 28115366 PMC5374731

[123] Moore PS, Chang Y. Why do viruses cause cancer? Highlights of the first century of human tumour virology. Nat Rev Cancer. (2010) 10:878–89. doi: 10.1038/nrc2961 21102637 PMC3718018

[124] Chen X, Kost J, Sulovari A, Wong N, Liang WS, Cao J, A virome-wide clonal integration analysis platform for discovering cancer viral etiology. Genome Res. (2019) 29:819–30. doi: 10.1101/gr.242529.118 30872350 PMC6499315

[125] Cao S, Wendl MC, Wyczalkowski MA, Wylie K, Ye K, Jayasinghe R, Divergent viral presentation among human tumors and adjacent normal tissues. Sci Rep. (2016) 6:28294. doi: 10.1038/srep28294 27339696 PMC4919655

[126] Romanczuk H, Howley PM. Disruption of either the E1 or the E2 regulatory gene of human papillomavirus type 16 increases viral immortalization capacity. Proc Natl Acad Sci U S A. (1992) 89:3159–63. doi: 10.1073/pnas.89.7.3159 1313584 PMC48824

[127] Cheung JL, Lo KW, Cheung TH, Tang JW, Chan PK. Viral load, E2 gene disruption status, and lineage of human papillomavirus type 16 infection in cervical neoplasia. J Infect Dis. (2006) 194:1706–12. doi: 10.1086/509622 17109343

[128] Shuda M, Feng H, Kwun HJ, Rosen ST, Gjoerup O, Moore PS, T antigen mutations are a human tumor-specific signature for Merkel cell polyomavirus. Proc Natl Acad Sci U S A. (2008) 105:16272–7 doi: 10.1073/pnas.0806526105 18812503 PMC2551627

[129] Naipauer J, Salyakina D, Journo G, Rosario S, Williams S, Abba M, High-throughput sequencing analysis of a “hit and run” cell and animal model of KSHV tumorigenesis. PLoS Pathog. (2020) 16:e1008589. doi: 10.1371/journal.ppat.1008589 32603362 PMC7357787

[130] Cancian L, Hansen A, Boshoff C. Cellular origin of Kaposi’s sarcoma and Kaposi’s sarcoma-associated herpesvirus-induced cell reprogramming. Trends Cell Biol. (2013) 23:421–32. doi: 10.1016/j.tcb.2013.04.001 23685018

[131] Iacovides D, Michael S, Achilleos C, Strati K. Shared mechanisms in stemness and carcinogenesis: lessons from oncogenic viruses. Front Cell Infect Microbiol. (2013) 3:66. doi: 10.3389/fcimb.2013.00066 24400225 PMC3872316

[132] Hibner U, Gregoire D. Viruses in cancer cell plasticity: the role of hepatitis C virus in hepatocellular carcinoma. Contemp Oncol. (2015) 19:A62–67. doi: 10.5114/wo.2014.47132 PMC4322526 25691824

[133] Xiang T, Lin YX, Ma W, Zhang HJ, Chen KM, He GP, Vasculogenic mimicry formation in EBV-associated epithelial malignancies. Nat Commun. (2018) 9:5009. doi: 10.1038/s41467-018-07308-5 30479336 PMC6258759

[134] Reid P, Marcu LG, Olver I, Moghaddasi L, Staudacher AH, Bezak E. Diversity of cancer stem cells in head and neck carcinomas: the role of HPV in cancer stem cell heterogeneity, plasticity and treatment response. Radiother Oncol. (2019) 135:1–12. doi: 10.1016/j.radonc.2019.02.016 31015153

[135] Shen S, Clairambault J. Cell plasticity in cancer cell populations. F1000Res. (2020) 9:F1000. doi: 10.12688/f1000research.24803.1 PMC7309415 32595946

[136] Nijman SMB. Perturbation-driven entropy as a source of cancer cell heterogeneity. Trends Cancer. (2020) 6:454–61. doi: 10.1016/j.trecan.2020.02.016 32460001

[137] Chang Y, Moore PS, Weiss RA. Human oncogenic viruses: nature and discovery. Philos Trans R Soc Lond B Biol Sci. (2017) 372:20160264. doi: 10.1098/rstb.2016.0264 28893931 PMC5597731

[138] Dhawan A, Scott J, Sundaresan P, Veness M, Porceddu S, Hau E, Role of gene signatures combined with pathology in classification of oropharynx head and neck cancer. Sci Rep. (2020) 10:10226. doi: 10.1038/s41598-020-66983-x 32576885 PMC7311543

[139] Bhatia K, Goedert JJ, Modali R, Preiss L, Ayers LW. Merkel cell carcinoma subgroups by Merkel cell polyomavirus DNA relative abundance and oncogene expression. Int J Cancer. (2010) 126:2240–6. doi: 10.1002/ijc.24676 19551862 PMC2908249

[140] Ho DW, Tsui YM, Chan LK, Sze KM, Zhang X, Cheu JW, Single-cell RNA sequencing shows the immunosuppressive landscape and tumor heterogeneity of HBV-associated hepatocellular carcinoma. Nat Commun. (2021) 12:3684. doi: 10.1038/s41467-021-24010-1 34140495 PMC8211687

[141] Krause J, von Felden J, Casar C, Frundt TW, Galaski J, Schmidt C, Hepatocellular carcinoma: intratumoral EpCAM-positive cancer stem cell heterogeneity identifies high-risk tumor subtype. BMC Cancer. (2020) 20:1130. doi: 10.1186/s12885-020-07580-z 33225916 PMC7682021

[142] Yamagishi M, Kubokawa M, Kuze Y, Suzuki A, Yokomizo A, Kobayashi S, Chronological genome and single-cell transcriptome integration characterizes the evolutionary process of adult T cell leukemia-lymphoma. Nat Commun. (2021) 12:4821. doi: 10.1038/s41467-021-25101-9 34376672 PMC8355240

[143] Messinger JE, Dai J, Stanland LJ, Price AM, Luftig MA. Identification of host biomarkers of Epstein-Barr virus latency IIb and latency III. MBio. (2019) 10. doi: 10.1128/mBio.01006-19 PMC6606803 31266868

[144] Yi M, Cai J, Li J, Chen S, Zeng Z, Peng Q, Rediscovery of NF-kappaB signaling in nasopharyngeal carcinoma: how genetic defects of NF-kappaB pathway interplay with EBV in driving oncogenesis? J Cell Physiol. (2018) 233:5537–49. doi: 10.1002/jcp.26410 29266238

[145] Bailey C, Shoura MJ, Mischel PS, Swanton C. Extrachromosomal DNA-relieving heredity constraints, accelerating tumour evolution. Ann Oncol. (2020) 31:884–93. doi: 10.1016/j.annonc.2020.03.303 32275948

[146] Manners O, Murphy JC, Coleman A, Hughes DJ, Whitehouse A. Contribution of the KSHV and EBV lytic cycles to tumourigenesis. Curr Opin Virol. (2018) 32:60–70. doi: 10.1016/j.coviro.2018.08.014 30268927 PMC6259586

[147] Raab-Traub N, Flynn K. The structure of the termini of the Epstein-Barr virus as a marker of clonal cellular proliferation. Cell. (1986) 47:883–9. doi: 10.1016/0092-8674(86)90803-2 3022942

[148] Pathmanathan R, Prasad U, Sadler R, Flynn K, Raab-Traub N. Clonal proliferations of cells infected with Epstein-Barr virus in preinvasive lesions related to nasopharyngeal carcinoma. N Engl J Med. (1995) 333:693–8 doi: 10.1056/NEJM199509143331103 7637746

[149] Feederle R, Neuhierl B, Bannert H, Geletneky K, Shannon-Lowe C, Delecluse HJ. Epstein-Barr virus B958 produced in 293 cells shows marked tropism for differentiated primary epithelial cells and reveals interindividual variation in susceptibility to viral infection. Int J Cancer. (2007) 121:588–94. doi: 10.1002/ijc.22727 17417777

[150] Tsang CM, Yip YL, Lo KW, Deng W, To KF, Hau PM, Cyclin D1 overexpression supports stable EBV infection in nasopharyngeal epithelial cells. Proc Natl Acad Sci U S A. (2012) 109:E3473–3482. doi: 10.1073/pnas.1202637109 23161911 PMC3528537

[151] Tsao SW, Tsang CM, Lo KW. Epstein-Barr virus infection and nasopharyngeal carcinoma. Philos Trans R Soc Lond B Biol Sci. (2017) 372. doi: 10.1098/rstb.2016.0270 PMC5597737 28893937

[152] Miller CA, McMichael J, Dang HX, Maher CA, Ding L, Ley TJ, Visualizing tumor evolution with the fishplot package for R. BMC Genomics. (2016) 17:880. doi: 10.1186/s12864-016-3195-z 27821060 PMC5100182

[153] Yu G, Hsu WL, Coghill AE Yu KJ, Wang CP, Lou PJ, Whole-Exome sequencing of nasopharyngeal carcinoma families reveals novel variants potentially involved in nasopharyngeal carcinoma. Sci Rep. (2019) 9:9916. doi: 10.1038/s41598-019-46137-4 31289279 PMC6617453

[154] Chaigne-Delalande B, Li FY, O’Connor GM, Lukacs MJ, Jiang P, Zheng L, Mg2+ regulates cytotoxic functions of NK and CD8T cells in chronic EBV infection through NKG2D. Science. (2013) 341:186–91. doi: 10.1126/science.1240094 23846901 PMC3894782

[155] Dhalla F, Murray S, Sadler R, Chaigne-Delalande B, Sadaoka T, Soilleux E, Identification of a novel mutation in MAGT1 and progressive multifocal leucoencephalopathy in a 58-year-old man with XMEN disease. J Clin Immunol. (2015) 35:112–8. doi: 10.1007/s10875-014-0116-2 25504528 PMC6328310

[156] Nakhoul H, Lin Z, Wang X, Roberts C, Dong Y, Flemington E. High-Throughput sequence analysis of peripheral T-Cell lymphomas indicates subtype-specific viral gene expression patterns and immune cell microenvironments. mSphere. (2019) 4:e00248–19. doi: 10.1128/mSphere.00248-19 31292228 PMC6620372

[157] Chau SL, Tong JH, Chow C, Kwan JS, Lung RW, Chung LY, Distinct molecular landscape of Epstein-Barr Virus associated pulmonary lymphoepithelioma-like carcinoma revealed by genomic sequencing. Cancers. (2020) 12:2065. doi: 10.3390/cancers12082065 PMC7463519 32726920

[158] Schiffman M, Wentzensen N. Human papillomavirus infection and the multistage carcinogenesis of cervical cancer. Cancer Epidemiol Biomarkers Prev. (2013) 22:553–60. doi: 10.1158/1055-9965.EPI-12-1406 23549399 PMC3711590

[159] Spurgeon ME, Liem A, Buehler D, Cheng J, DeCaprio JA, Lambert PF. The Merkel cell polyomavirus T antigens function as tumor promoters in murine skin. Cancers. (2021) 13:222. doi: 10.3390/cancers13020222 PMC7827793 33435392

[160] Izaki M, Yasunaga JI, Nosaka K, Sugata K, Utsunomiya H, Suehiro Y, *In vivo* dynamics and adaptation of HTLV-1-infected clones under different clinical conditions. PLoS Pathog. (2021) 17:e1009271. doi: 10.1371/journal.ppat.1009271 33524072 PMC7877780

[161] Sartorius K, Swadling L, An P, Makarova J, Winkler C, Chuturgoon A, The multiple roles of hepatitis b virus X Protein (HBx) dysregulated microRNA in hepatitis B virus-associated hepatocellular carcinoma (HBV-HCC) and immune pathways. Viruses. (2020) 12:746. doi: 10.3390/v12070746 PMC7412373 32664401

[162] Li Q, Wennborg A, Aurell E, Dekel E, Zou JZ, Xu Y, Dynamics inside the cancer cell attractor reveal cell heterogeneity, limits of stability, and escape. Proc Natl Acad Sci U S A. (2016) 113:2672–7. doi: 10.1073/pnas.1519210113 26929366 PMC4790994

[163] Ladewig J, Koch P, Brustle O. Leveling Waddington: the emergence of direct programming and the loss of cell fate hierarchies. Nat Rev Mol Cell Biol. (2013) 14:225–36. doi: 10.1038/nrm3543 23486282

[164] Waddington CH, Robertson E. Selection for developmental canalisation. GenetRes. (1966) 7:303–12. doi: 10.1017/S0016672300009769 5940870

[165] Chen H, He X. The convergent cancer evolution toward a single cellular destination. Mol Biol Evol. (2016) 33:4–12. doi: 10.1093/molbev/msv212 26464125

[166] Urban EA, Johnston RJ Jr. Buffering and amplifying transcriptional noise during cell fate specification. Front Genet. (2018) 9:591. doi:10.3389/fgene.2018.00591 30555516 PMC6282114

[167] Huang S, Ernberg I, Kauffman S. Cancer attractors: a systems view of tumors from a gene network dynamics and developmental perspective. Semin Cell Dev Biol. (2009) 20:869–76. doi: 10.1016/j.semcdb.2009.07.003 19595782 PMC2754594

[168] Scott J, Marusyk A. Somatic clonal evolution: a selection-centric perspective. Biochim Biophys Acta Rev Cancer. (2017) 1867:139–50. doi: 10.1016/j.bbcan.2017.01.006 28161395

[169] Westrich JA, Warren CJ, Pyeon D. Evasion of host immune defenses by human papillomavirus. Virus Res. (2017) 231:21–33. doi: 10.1016/j.virusres.2016.11.023 27890631 PMC5325784

[170] Jenkinson G, Pujadas E, Goutsias J, Feinberg AP. Potential energy landscapes identify the information-theoretic nature of the epigenome. Nat Genet. (2017) 49:719–29. doi: 10.1038/ng.3811 28346445 PMC5565269

[171] Chan KCA, Woo JKS, King A, Zee BCY, Lam WKJ, Chan SL, Analysis of plasma Epstein-Barr virus DNA to screen for nasopharyngeal cancer. N Engl J Med. (2017) 377:513–22. doi: 10.1056/NEJMoa1701717 28792880

[172] Ji MF, Sheng W, Cheng WM, Ng MH, Wu BH, Yu X, Incidence and mortality of nasopharyngeal carcinoma: interim analysis of a cluster randomized controlled screening trial (PRO-NPC-001) in southern China. Ann Oncol. (2019) 30:1630–7. doi: 10.1093/annonc/mdz231 31373615

[173] Zhang Y, Li Y, Li T, Shen X, Zhu T, Tao Y, Genetic load and potential mutational meltdown in cancer cell populations. Mol Biol Evol. (2019) 36:541–52. doi: 10.1093/molbev/msy231 30649444

